# Dataset for field experiments analyzing discrimination in amateur soccer

**DOI:** 10.1016/j.dib.2021.107751

**Published:** 2021-12-23

**Authors:** Cornel Nesseler, Carlos Gomez-Gonzalez, Helmut Dietl, Christoph Halser

**Affiliations:** a Norwegian University of Science and Technology, Trondheim, Norway; b University of Zurich, Zurich, Switzerland

**Keywords:** Sport science, Experiments, Discrimination, Migration, Integration, Social activities, RCT, Amateur soccer

## Abstract

This paper presents data of field experiments that analyze discrimination in amateur soccer. The studies created fake accounts and asked amateur soccer coaches to come for a trial practice. The fake accounts had either a native- or a foreign-sounding name. The dataset is based on three published studies that analyzed discrimination in 23 countries. The dataset contains 24,915 observations and several variables that are interesting for further research. This data can be used to compare discrimination in amateur soccer with discrimination in diverse fields, such as migration, economics, or political science. For a more detailed discussion, please see the published articles.

## Specifications Table

**Table:** 

Subject area	Sport Science
More specific subject area	Discrimination in Sport
Type of data	Text files and Stata .dta format
How the data were acquired	Field experiments
Data format	Raw and Analyzed (in published studies)
Description of data collection	Data was retrieved from the authors of the studies and merged into one dataset. Data was merged with Stata queries. The labeling ensures that readers can analyze the data without intensive knowledge of the data
Data source location	University of Zurich, Zurich, Switzerland
Data accessibility	Data is within this article and on HarvardDataverse, doi: 10.7910/DVN/TBULFV
Related research article	1. Nesseler, C., Gomez-Gonzalez, C., & Dietl, H. (2019). What's in a name? Measuring access to social activities with a field experiment. Palgrave Communications, 5(1), 1-7. https://www.nature.com/articles/s41599-019-0372-0 2. Dietl, H. M., Gomez-Gonzalez, C., Moretti, P., & Nesseler, C. (2021). Does persistence pay off? Accessing social activities with a foreign-sounding name. Applied Economics Letters, 28(10), 881-885. https://www.tandfonline.com/doi/full/10.1080/13504851.2020.1784381 3. Gomez-Gonzalez, C., Nesseler, C., & Dietl, H. M. (2021). Mapping discrimination in Europe through a field experiment in amateur sport. Humanities and Social Sciences Communications, 8(1), 1-8. https://www.nature.com/articles/s41599-021-00773-2

## Value of the Data

• The dataset provides future research with the possibility to analyze discrimination in amateur soccer and examine within- and between-country differences
• The dataset contains response rates to inquires in amateur soccer, which could be compared with discrimination outcomes in other social domains (e.g., housing, labor, or sharing economy)
• The dataset shows specific foreign groups that suffer from discrimination (e.g., people with a Turkish-sounding name in Germany or with a German-sounding name in Denmark). This makes it possible to compare the extent of discrimination of specific foreign groups in different countries.
• The dataset contains information to validate or contradict the usefulness of covariates that are thought to have an influence on discrimination (e.g., share of foreign-born population or share of right-wing voters)
• Researchers in similar field experiments can append their data.
• Educators can use the dataset to give students a broad understanding of field experiments.

## 1 Data Description

The dataset presented in this paper combines the dataset of three published papers. All papers analyze discrimination in amateur soccer from a similar perspective [1] examine discrimination towards people with either a Swiss-French, Swiss-German, Swiss-Italian, Eastern European, Turkish, or Arabian-sounding name. The authors contact amateur soccer clubs in Switzerland with fake accounts using the following email ([1], p.3):

**Table 1:** Variables description.

Variable	Type	Description
*Country*	Categorical	Name of the country in which the experiment took place
*ForeignGroup1*	Categorical	Name (country) of the first foreign group that contacted amateur football clubs
*ForeignGroup2*	Categorical	Name (country) of the second foreign group that contacted amateur football clubs
*ForeignGroup3*	Categorical	Name (country) of the third foreign group that contacted amateur football clubs
*id*		A unique code for each row
*League*	Categorical	League in which the club played that was contacted in the experiment
*Native*	Integer	Sender who contacted the club had either a native-sounding or foreign-sounding name
*Num_ForeignGroup1*	Integer	Numeric (binary) variable of ForeignGroup1
*Num_ForeignGroup2*	Integer	Numeric (binary) variable of ForeignGroup2
*Num_ForeignGroup3*	Integer	Numeric (binary) variable of ForeignGroup3
*Resilience*	Integer	Reminder email sent in the Dietl et al. (2020)
Response*	Integer	Response of the club. The response can either be negative, positive, or positive with further inquiries
*Sender*	Categorical	Name (country) of the sender
*SimpleResponse*	Integer	Variable *Response* simplified into negative or positive
*SoccerRegion*⁎⁎	Categorical	Region as defined by soccer federations/leagues
*StudyYear*	Numeric	Year in which the study was published
*Study*	Numeric	Unique number for each study
*SwissFrenchNames*	Categorical	Name of the sender was Swiss-French
*SwissGermanNames*	Categorical	Name of the sender was Swiss-German
*SwissItalianNames*	Categorical	Name of the sender was Swiss-Italian

⁎ This data is not available for Hungary, England, Portugal, Romania, and the experiment [2].

⁎⁎ This data is not available for [2].

*Subject: Trial practice*

*Hello,*

*I would like to take part in a trial training session with your team. I have already played at a similar level. Could I come for a trial training session?*

*Many thanks*

*Name*

The authors then categorize the response of the coach into negative, positive, or positive with additional inquiries. This is captured in the variable *Response.* All variables are listed in Table 1. Afterwards, the authors simplify the variable into either a positive response or a negative response (see variable *SimpleResponse*).

Dietl et al. [2] replicate the study of [1]. However, they contact a subsample of the amateur soccer clubs that did not respond to the first email again. They call this process “resilience” (the variable has the same name).

Gomez-Gonzalez et al. [3] extend the study of [1] and reach amateur soccer clubs in 22 European countries. They contact the clubs in each country with either a native-sounding name or a name from one of the three largest foreign-sounding groups. The countries for all three studies are listed in Table 2.

**Table 2:** Overview of countries.

Country	Obs.	%	Foreign-sounding groups
Austria	1,840	7.39	Serbia, Turkey, Bosnia-and-Herzegovinia
Belgium	663	2.66	Italy, Morocco, Poland
Croatia	447	1.79	Germany, Italy, China
Czech Republic	1,598	6.41	Ukraine, Vietnam, Russia
Denmark	1,135	4.56	Turkey, Poland, Germany
England	1,527	6.13	Poland, India, Italy
Finland	536	2.15	Estonia, Russia, Somalia
France	1,847	7.41	Portugal, Algeria, Morocco
Germany	1,681	6.75	Turkey, Italy, Poland
Greece	437	1.75	Albania, Bulgaria, Romania
Hungary	798	3.20	Romania, Germany, Ukraine
Ireland	308	1.24	Poland, Lithuania, Latvia
Italy	1,463	5.87	Romania, Albania, Morocco
Netherlands	715	2.87	Turkey, Morocco, Indonesia
Norway	1,000	4.01	Poland, Germany, Lithuania
Poland	1,312	5.27	Ukraine, Germany, Belarus
Portugal	791	3.17	Ukraine, Angola, Romania
Romania	493	1.98	Turkey, China, Germany
Russian Federation	1,143	4.59	Kazakhstan, Uzbekistan, Azerbaijan
Serbia	383	1.54	Hungary, Slovakia, Albania
Spain	1,410	5.66	Morocco, Romania, England
Sweden	1,493	5.99	Finland, Iraq, Poland
Switzerland	1,895	7.61	Eastern Europe, Turkey, Arabia [1]; Serbia/Croatia, Turkey, Arabia [2]

Total	24,915	100	

The variables *ForeignGroup1, ForeignGroup2, ForeignGroup3* show the country of origin for the foreign group for each country (e.g., Ukrainian-, Vietnamese-, and Russian-sounding names in the Czech Republic).

## 2 Experimental Design, Materials and Methods

**Fig. 1:**
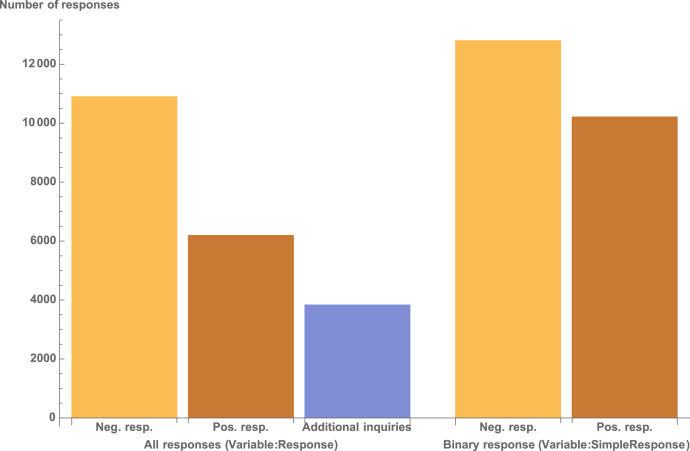
Response rate overview.

The initial data was gathered through three field experiments. Parts of the data were made public after the publication of each study. We received the missing data from the authors of the studies. However, still not all studies had the information for the same variables. For example, the data for the original unmodified Response is not available for [2]. Additionally, this data is missing for a few countries in [3] (see footnote for Table 1). The simplified response rate is available for all countries (see Fig. 1).

We then labeled and named each variable in each study in accordance with each other. It is important to note that the foreign groups for each country are specifically listed in the dataset but also available in a numeric format. This ensures that future research can focus on specific foreign groups but also on specific countries. This is similar for the variable *Sender*. While the variable *Native* shows if a request was sent from a native- or foreign-sounding name, *Sender* shows the nationality of the person that sent the request.

## Ethics Statement

All experiment received an ethical approval from the University of Zurich. [1] had the approval date 14.07.2017 (no IRB number available), [2] had the IRB #2019-053, and [3] had the IRB #2019–006.

## CRediT Author Statement

All authors contributed equally to this study and share the responsibility for its content.

## Declaration of Competing Interest

The authors declare no competing interests.

## Appendix Supplementary materials

Image, application 1

Image, application 2

Image, application 3
